# Achieving affective human–virtual agent communication by enabling virtual agents to imitate positive expressions

**DOI:** 10.1038/s41598-020-62870-7

**Published:** 2020-04-06

**Authors:** Takashi Numata, Hiroki Sato, Yasuhiro Asa, Takahiko Koike, Kohei Miyata, Eri Nakagawa, Motofumi Sumiya, Norihiro Sadato

**Affiliations:** 1 Center for Exploratory Research, Research & Development Group, Hitachi, Ltd., Hatoyama, Saitama, 350-0395 Japan; 2 Department of Bioscience and Engineering, Shibaura Institute of Technology, Saitama, Saitama, 337-8570 Japan; 3 Division of Cerebral Integration, Department of System Neuroscience, National Institute for Physiological Sciences, Okazaki, Aichi 444-8585 Japan

**Keywords:** Cognitive control, Prefrontal cortex, Agency, Empathy, Quality of life

## Abstract

Affective communication, communicating with emotion, during face-to-face communication is critical for social interaction. Advances in artificial intelligence have made it essential to develop affective human–virtual agent communication. A person’s belief during human–virtual agent interaction that the agent is a computer program affects social-cognitive processes. Whether this belief interferes with affective communication is an open question. We hypothesized that the imitation of a positive emotional expression by a virtual agent induces a positive emotion, regardless of the belief. To test this hypothesis, we conducted an fMRI study with 39 healthy volunteers, who were made to believe that a virtual agent was either a person or a computer. They were instructed to smile, and immediately afterwards, the virtual agent displayed a positive, negative, or neutral expression. The participants reported a positive emotion only when their smile was imitated by the agent’s positive expression regardless of their belief. This imitation activated the participants’ medial prefrontal cortex and precuneus, which are involved in anthropomorphism and contingency, respectively. These results suggest that a positive congruent response by a virtual agent can overcome the effect of believing that the agent is a computer program and thus contribute to achieving affective human–virtual agent communication.

## Introduction

Communicating with emotion, i.e., affective communication, plays an important role in inducing empathy and enhancing human bonding^1–3^. With advances in artificial intelligence, virtual agents are starting to play an active role in various fields such as information presentation, sales, training, education, and healthcare^4–10^. To help induce a positive emotion and thereby enhance social bonding in human–virtual agent interaction, affective communication between people and virtual agents has become important^11^.

To achieve the affective communication between people and virtual agents, one of the important issues is a person’s belief. A person’s belief during human–virtual agent interaction that the agent is a computer program, not a human agent, affects social-cognitive processes^12,13^. For example, Caruana *et al*. reported that subjective ratings of an agent as pleasant and cooperative was lower and saccadic reaction time was longer during a joint attention task in the virtual agent condition than those in the human (virtual avatar) condition, with these conditions being regulated by agency manipulation^13^. Thus, the person’s belief about the agent, “agency belief,” is an important aspect of affective communication with virtual agents.

As a means to induce a positive emotion in a person interacting with a virtual agent controlled by a computer program, several researchers have proposed having virtual agents imitate human behavior^14–17^. Mimicry (automatic imitation^18^) of the human partner’s behaviors is known to elicit positive emotions in human communication^19^. This means that it might be useful to find ways to suppress the effect of agency belief and instead induce positive emotions in the human partner. This realization led to the development of virtual agents with realistic human features and functions for imitating human behavior^14–17^. However, these imitation functions do not always induce significant positive emotions in people^14,17^. This is because the psychophysiological mechanisms of being imitated are still unclear. Toward the understanding of the psychophysiological mechanisms, previous studies evaluated the effects of being imitated on the basis of measured brain activity^18,20–22^. Understanding the underlying psychophysiological mechanisms when a person is being imitated by a virtual agent should help to identify the key factors inducing a positive emotion, and this should contribute to the development of virtual agent behaviors that effectively counteract the effect of agency belief.

In this study, we assumed that one of the key factors inducing a positive emotion is the imitation of positive emotional expressions, whereas previous studies in regard to human–virtual agent interaction focused mainly on imitation behaviors without considering emotional expression^12–17,21–24^. Although there have been a few studies that considered emotional expression^25,26^, the mimickers were human. None of the studies considered imitation of emotional expressions by a virtual agent. Mimicry of emotional expressions is done automatically in human-to-human communication and plays an important role in achieving affective communication^27–29^. In addition, in human-to-human communication, the mimicry of facial emotional expressions can promote the liking of the interaction partner^30^. Specifically, the mimicry of a positive expression is a robust response in people^26^. Moreover, positive expressions themselves engender positive affect and/or positive impressions of the expressor^31,32^, whereas negative expressions themselves engender negative affect and/or negative impressions^33^. Therefore, the mimicry of a positive expression is a natural and robust response, and thus should be highly effective for inducing a positive emotion. Given these findings, we hypothesized that the imitation of a positive emotional expression helps make the response of the virtual agent seem natural and robust, like that of a person, and thereby induce a positive emotion in the human partner, regardless of whether he or she believes that the agent is a person or a computer. In other words, we expected that the imitation of a positive expression would help anthropomorphize the virtual agent. Anthropomorphism is the tendency to imbue the real or imagined behavior of non-human agents with humanlike characteristics, motivations, intentions, and emotions^34^, and thus should be an effective factor in inducing a positive emotion and thereby enhance social bonding in human–virtual agent interaction. We used functional magnetic resonance imaging (fMRI) to investigate the psychophysiological mechanisms related to the imitation of a positive expression. In particular, since the brain region that appears most centrally involved in anthropomorphism is the medial prefrontal cortex^35^, evaluation of the activations in that region should help clarify whether the virtual agent’s imitation of a positive expression helps induce an anthropomorphic feeling about the virtual agent.

To test our hypothesis and to better understand the psychophysiological mechanisms of being imitated by virtual agents, we conducted an fMRI study with 39 healthy volunteer participants. To focus on the effect of positive congruent responses by virtual agents and suppress the effects of appearance similarity with the participant’s face and behavioral similarity with the participant’s smile, we used a non-human virtual agent. Facial features are known to play an important role in processing emotional facial expressions^36^. For example, a person’s emotional recognition of expressions made by a non-human agent with a non-proportionally sized facial features to human and synthetic human facial features was worse than for those made by humans and synthetic humans^37^. This means that the facial disimilarity of non-human virtual agents strongly affects emotional expressions. Therefore, rather than using virtual agents with realistic human features as was done in previous studies^14–17^, we used a non-human virtual agent with dynamically formed expressions.

**Figure 1:**
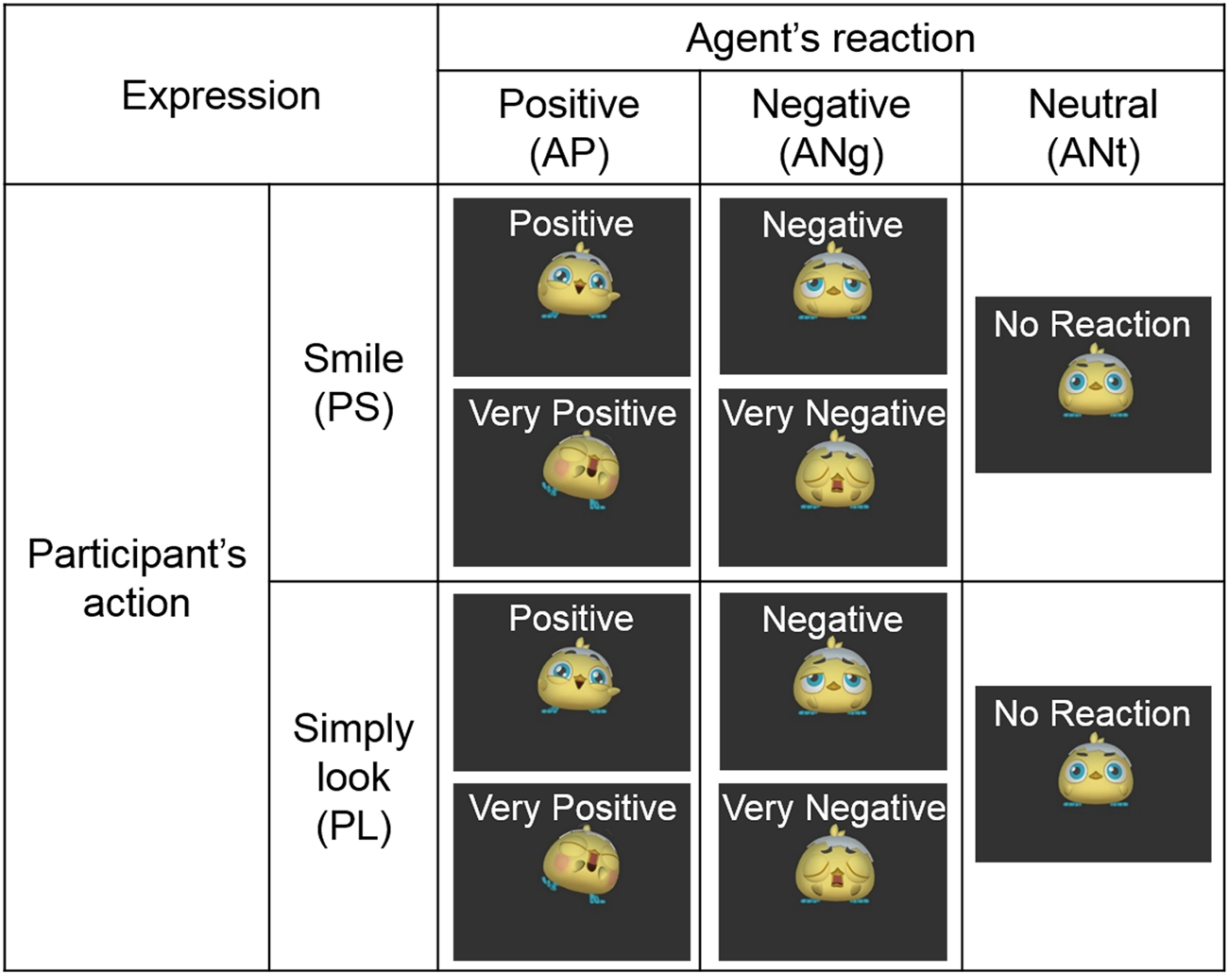
Experimental task. Participants were instructed either to smile (PS) or to simply look at the virtual agent (PL); immediately afterwards, the virtual agent displayed a positive expression (AP), a negative expression (ANg), or a neutral expression (ANt). The timing and strength (positive or very positive, and negative or very negative) of the virtual agent’s expression depended on the degree of the participant’s smile.

**Figure 2:**
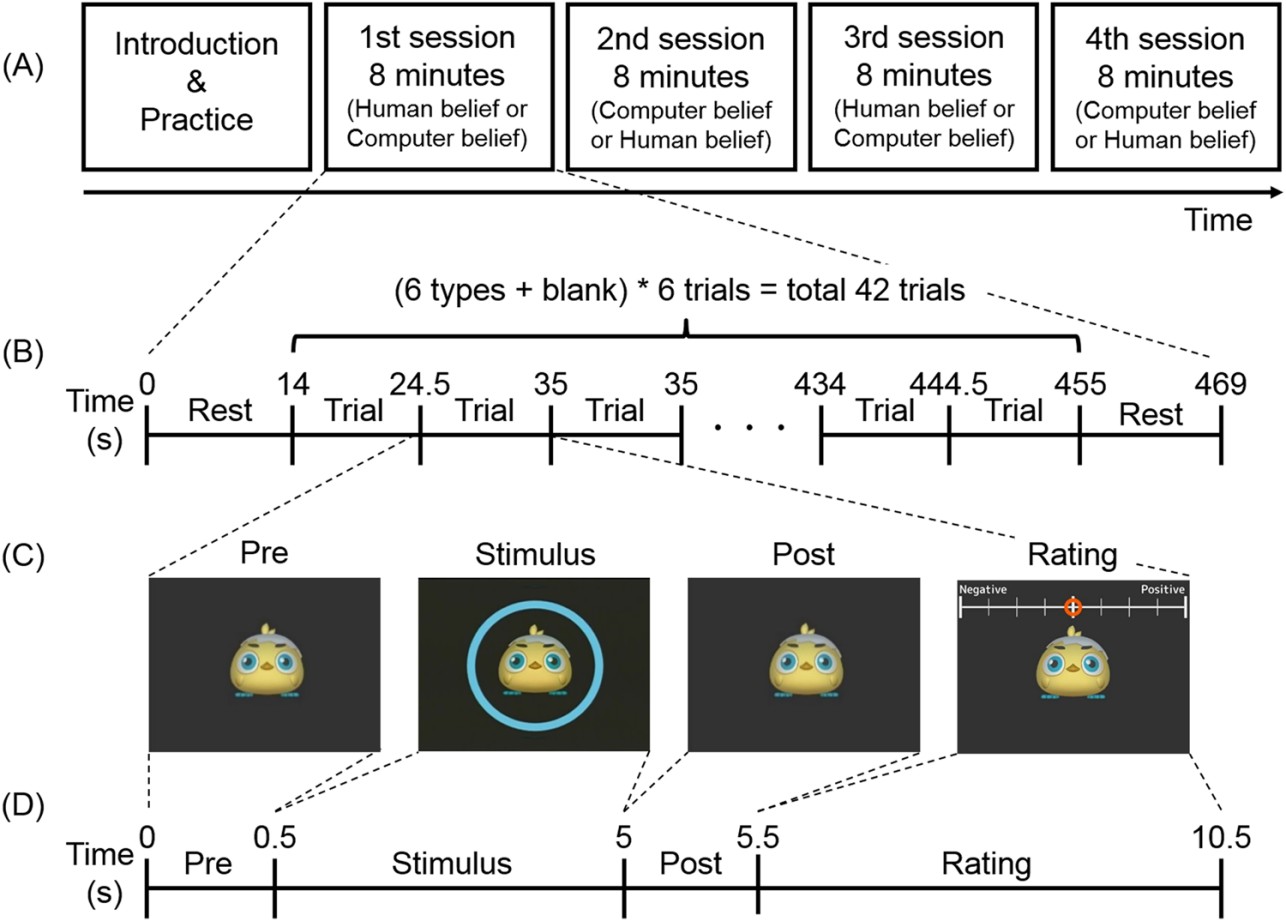
Experimental schematic. (**A**) Experiment consisted of four sessions, each lasting about eight minutes. Two were under the human belief condition with agency belief set, and the other two were under the computer belief condition with agency belief set. (**B**) Each session consisted of 42 trials: 6 trials of 6 task types and 6 blank trials. Pre- and post-trial rest periods were each set to 14 s, and each trial was set to 10.5 s. (**C**) A visual stimulus (a chick-type non-human virtual agent) was presented throughout each session. Participants were told to smile or simply look at the virtual agent when they saw a blue circle or pink square around the agent (stimulus phase), followed by the expression of the agent. After this facial interaction, participants were asked to rate their feeling on a 9-point scale (from −4 to 4). (**D**) Each trial consisted of a pre-period (0.5 s), a stimulus period (4.5 s), a post period (0.5 s), and a rating period (5.0 s).

The participants performed a facial interaction task with a non-human virtual agent in the form of a chick. They were instructed either to smile or to simply look at the chick. Immediately afterwards, the chick displayed a positive expression, a negative expression, or a neutral reaction (Fig. 1). The effect of their smile being imitated of their smile was evaluated (Table 1). The agent’s expressions were triggered by the participant smiling, and their strength reflected the degree of the participant’s smile in the smile condition. Each performance of the task was followed by a subjective rating by the participant of his or her current feeling (Fig. 2). The experiment was conducted in a human belief condition and in a computer belief condition; that is, the participant was made to believe that the agent was a person or a computer.

**Table 1:** Experimental conditions for contrast of interest.

Participant’s action	Smile (PS)			Simply look (PL)		
Agent’s reaction	Positive (AP)	Negative (ANg)	Neutral (ANt)	Positive (AP)	Negative (ANg)	Neutral (ANt)
Imitated smile effect	2	−1	−1	−2	1	1

Participants were instructed to smile (participant smile; PS) or simply look (participant look; PL) at the virtual agent. The agent then responded with a positive expression (AP), negative expression (ANg), or neutral expression (ANt). The effect of the participant’s smile being imitated was evaluated by using the contrast of (PS_AP − (PS_ANg + PS_ANt)/2) − (PL_AP − (PL_ANg + PL_ANt)/2).

## Results

### Behavioral results

**Figure 3:**
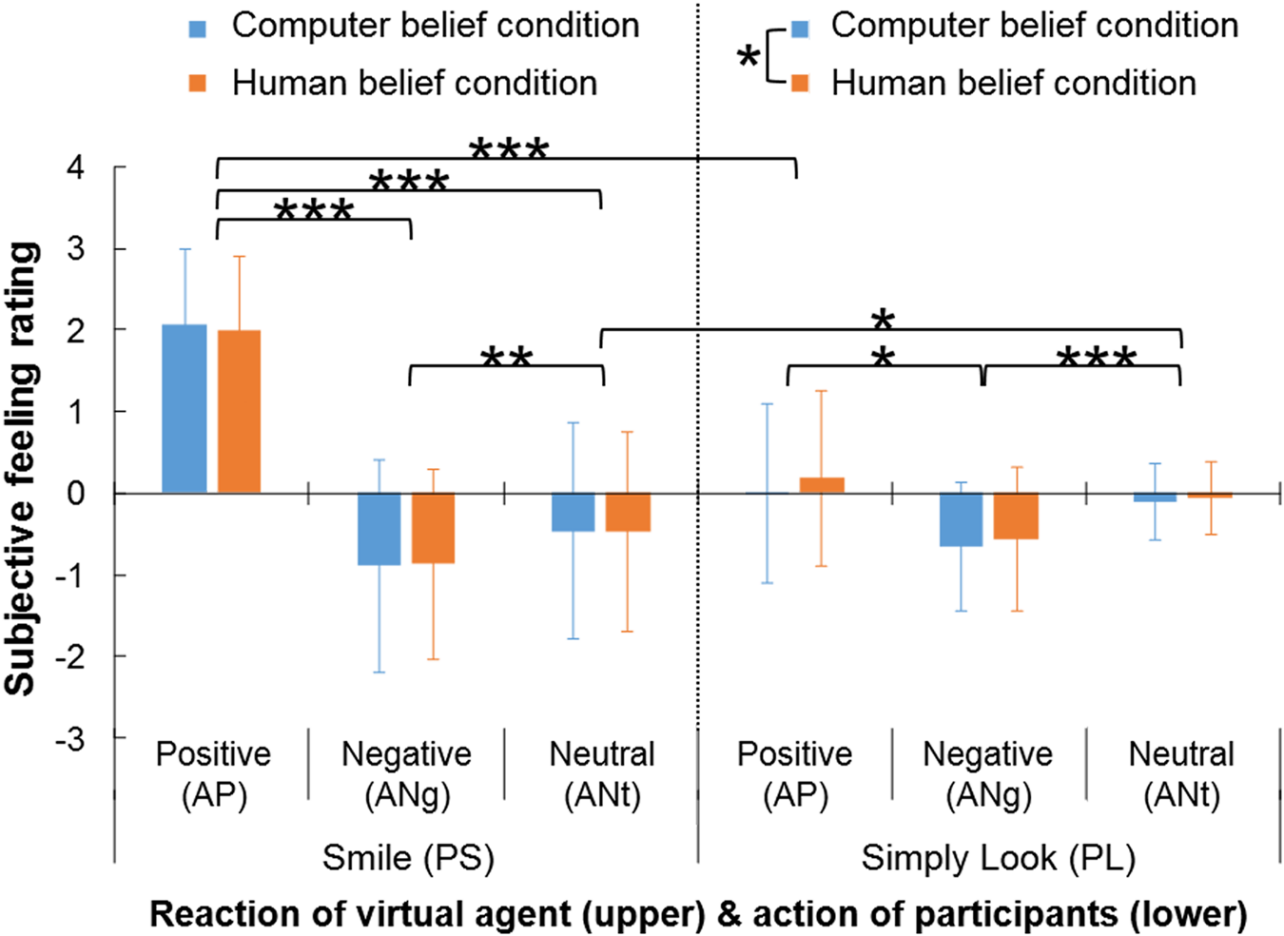
Behavioral results. Bars depicting subjective feeling ratings for each participant action (i.e., Smile, Simply Look), each agent reaction (i.e., Positive, Negative, Neutral), and each agency belief (i.e., Computer, Human). Error bars indicate standard deviation of mean in post hoc ANOVA test results: *indicates *p* < 0.05, **indicates *p* < 0.01, and ***indicates *p* < 0.001.

A three-way ANOVA was used to investigate the effects of participant action, agent reaction, and agency belief and their interactions on the participants’ subjective feeling (Fig. 3 and Supplementaly Table S1). The participants reported a positive emotion only when their smile was imitated by the agent, regardless of the agency belief.

In regard to the main effects, the ANOVA revealed that the main effects of participant action and agent reaction were significant [*F*(1, 38) = 17.737, *p* < 0.001, *η*^2^ = 0.318; *F*(2, 76) = 71.422, *p* < 0.001, *η*^2^ = 0.653, respectively] while that of agency belief was not significant [*F*(1, 38) = 1.620, *p* = 0.211, *η*^2^ = 0.041]. Thus, a participants’ subjective feeling differed depending on the participant’s action and agent’s reaction, and it did not differ depending on the agency belief.

In regard to their interactions, on the one hand, the ANOVA did not reveal a significant three-way interaction among participant action, agent reaction, and agency belief [*F*(1.624, 61.718) = 1.188, *p* = 0.304, *η*^2^ = 0.030] or a significant two-way interaction between agent reaction and agency belief [*F*(2, 76) = 0.355, *p* = 0.703, *η*^2^ = 0.009]. On the other hand, it did reveal a significant two-way interaction between participant action and agent reaction [*F*(1.199, 45.566) = 45.563, *p* < 0.001, *η*^2^ = 0.545]. More specifically, comparison among agent reactions in the participant smile (PS) condition revealed a significant effect of agent reaction [*F*(2, 76) = 86.672, *p* < 0.001, *η*^2^ = 0.695]. The post hoc test results revealed that the subjective feeling in the agent positive expression (AP) condition was significantly more positive than in the negative expression (ANg) and neutral expression (ANt) conditions (both *p* < 0.001). They also revealed that the subjective feeling in the ANg condition was significantly more negative than in the ANt condition (*p* = 0.002). Also, the results in the participant simply look (look; PL) condition revealed a significant effect of agent reaction [*F*(2, 76) = 8.970, *p* = 0.002, *η*^2^ = 0.191]. Furthermore, they revealed that the subjective feeling in the ANg condition was significantly more negative than those in the AP and ANt conditions (*p* = 0.010 and *p* < 0.001, respectively). In addition, comparison between participant actions in the AP condition revealed a significant effect of participant action [*F*(1, 38) = 79.218, *p* < 0.001, *η*^2^ = 0.676], meaning that the subjective feeling in the PS condition was significantly more positive than in the PL condition. Comparison between participant expressions in the ANt condition revealed a significant effect of participant action [*F*(1, 38) = 4.690, *p* = 0.037, *η*^2^ = 0.110], meaning that subjective feeling in the PS condition was significantly more negative than in the PL condition. Thus, the participants’ subjective feelings were more positive when their smile was imitated by the agent than it was in the other participant-action and agent-reaction conditions. Comparison also revealed a significant two-way interaction between participant action and agency belief [*F*(1, 38) = 6.462, *p* = 0.015, *η*^2^ = 0.145]. Comparison between the computer belief (CB) condition and the human belief (HB) condition revealed no significant effect of the belief condition in the PS condition [*F*(1, 38) =0.065, *p* = 0.800, *η*^2^ = 0.002], whereas it revealed a significant effect in the PL condition [*F*(1, 38) = 8.801, *p* = 0.005, *η*^2^ = 0.188]. Thus, the participants’ subjective feelings did not depend on the agency belief when the participants smiled while it depended on the belief when the participants simply looked at the agent.

### fMRI Results: Smile-imitation-condition-related activation

**Figure 4:**
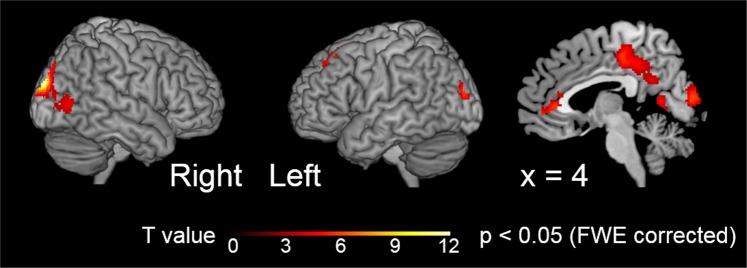
Significant clusters in the contrast of being imitated of the smile condition minus no being imitated conditions (PS_AP − (PS_ANg + PS_ANt)/2) − (PL_AP − (PL_ANg + PL_ANt)/2). Statistical threshold for cluster formation was set at *p* < 0.001, corrected for multiple comparisons over the whole brain. PS: participant smile, PL: participant simply looking, AP: agent positive expression, ANg: agent negative expression, ANt: agent neutral expression.

Significant brain regions specifically activated when the participant’s smile was imitated by the virtual agent, compared with other participant-action and agent-reaction conditions, were extracted by using fMRI data. The effect of smile imitation was evaluated by subtractions of the no imitation conditions in the participants’ smile conditions and participants’ no smile conditions from the smile imitation condition: (PS_AP − (PS_ANg + PS_ANt)/2) − (PL_AP − (PL_ANg + PL_ANt)/2) conditions. The effect of smile imitation was associated with significant activation in several regions of the brain, including the middle cingulate cortex (MCC), the precuneus, the cuneus, and the superior and middle occipital gyrus, the calcarine gyrus, the lingual gyrus, the superior and middle frontal gyrus, the anterior cingulate cortex (ACC), the superior medial gyrus, and the middle orbital gyrus (Table 2 and Fig. 4). A comparison between the computer belief condition and human belief condition under the smile-imitation condition did not reveal any significant cluster in regard to agency belief differences. In fact, there were no significant main effects of the two agency belief conditions, and there were no significant differences between when the participants were interacting with a person and when they were interacting with a computer program in the simply look condition. Our investigation of the parameteric effects of subjective feelings on brain activity did not reveal any brain regions that positevely tracked the level of subjective feeling.

**Table 2:** Significant clusters and their peak coordinates and anatomical regions in the contrast of being imitated of the smile condition minus no being imitated conditions (PS_AP-(PS_ANg + PS_ANt)/2) − (PL_AP−(PL_ANg + PL_ANt)/2).

Spatial extent test		MNI coordinates			T value	Hem	Anatomical Region
Cluster size (mm^3^)	P values	x	y	z			
52,728	P < 0.001	4	−24	48	5.99	R	Middle Cingulate Cortex
		−2	−40	40	5.89	L	Middle Cingulate Cortex
		−12	−56	14	5.90	L	Precuneus
		12	−96	12	11.28	R	Cuneus
		20	−92	24	12.82	R	Superior Occipital Gyrus
		−32	−86	18	5.86	L	Middle Occipital Gyrus
		18	−84	12	10.57	R	Calcarine Sulcus
		−8	−64	18	6.10	L	Calcarine Sulcus
		12	−72	−6	9.85	R	Lingual Gyrus
3,128	P < 0.001	−22	32	48	5.94	L	Superior Frontal Gyrus
		−22	22	52	4.47	L	Middle Frontal Gyrus
2,600	P < 0.001	4	32	14	4.11	R	Anterior Cingulate Cortex
		6	50	4	3.48	R	Superior Medial Gyrus
		2	48	−4	5.68	R	Middle Orbital Gyrus

Statistical threshold for cluster formation was set at *p* < 0.001 uncorrected, and for cluster-level family-wise error (FWE) corrected, it was set at *p* < 0.05. PS: participant smile, PL: participant simply looking, AP: agent positive expression, ANg: agent negative expression, ANt: agent neutral expression. Hem: hemisphere, R: right, L: left.

## Discussion

Since agency belief significantly affected the participant’s feeling when the participant simply looked at the virtual agent’s expression, the effect of agency belief should have been a significant factor in the experiment. However, consistent with our hypothesis, the imitation of a positive expression by the virtual agent induced a positive emotion in the participant that was not affected by agency belief. Therefore, a positive emotion could be induced even when the participants knew that the virtual agent was controlled by a computer program. These results suggest that a positive congruent response by a virtual agent can overcome the effect of agency belief and contribute to achieving affective human–virtual agent communication.

Whereas a relationship between the agency belief and the imitation of a positive expression was indicated, the effects of other factors should also be considered; further research is needed to clarify the relationship between the agency belief and the imitation of a positive expression. The key factors in suppressing the effect of agency belief can be identified by testing in various experimental conditions. For example, using a cover story that makes the participants think that the “correct” response by the virtual agent is the display of a negative expression in response to a positive expression by the participant would avoid the effect of decoding correctness. In addition, top-down versus bottom-up distinction would be helpful to deeply understand the effect of the imitation of a positive expression. Since the positive expressions were imitated only 33% of the time in the experiment, seeing the virtual agent positively and contingently engaged with the participant should have evoked bottom-up recognition. However, the participants could have expected that their smile would be imitated by the virtual agent, and the imitation of a positive expression could have evoked top-down recognition. Therefore, it is not clear whether the imitation of a positive expression simply suppressed the effect of agency belief or the effect of bottom-up recognition (the feeling of seeing the virtual agent positively and contingently engaged with the participants) suppressed the effect of top-down recognition (the belief that an agent was a computer program). The control of imitation frequency should thus be useful to evaluate the effects of top-down and bottom-up recognition when the virtual agent imitates a positive expression.

The participants reported negative emotions when their smile was followed by a negative expression by the agent although the temporal contingency was the same as for a positive expression by the agent. This finding does not match the results in a previous study, which found that action dissimilarity had no negative effect by using being imitated of neutral behavior^24^. This difference from the previous study indicates that positiveness should affect the emotions induced by imitation when the participant’s behavior includes emotional expression. This means that a positive congruent response by a virtual agent is important in inducing positive emotion in participants.

Rather than using a human virtual agents as used in previous studies^13,21^, we used a non-human virtual agent in the form of a creature with a face morphologically dissimilar to a human face. Our finding that being imitated by a non-human virtual agent can induce a positive emotion in participants indicates that appearance similarity between human and virtual agents is not an important factor. Alternatively, we speculate that an excessive and flexible expression results in a natural imitation response by the non-human virtual agent as well as human emotional mimicry, and this might contribute to a positive emotion even in the computer belief condition. Although further research is needed using various appearances and expressions of virtual agents, the feasibility of using virtual agents with divergent appearances and expressions for interacting with people is suggested by the results of this study.

The fMRI results showed that imitation of a positive expression activated the medial prefrontal cortex and the precuneus while no significant clusters were extracted by a comparison between agency belief conditions. Along with our hypothesis of psychophysiological mechanisms, there was indication of activation of the medial prefrontal cortex was extracted, which was suggested to be centrally involved in anthropomorphism by the results of a previous study^35^. Since the mimicry of emotional expressions is done automatically in human-to-human communication^27–29^, it is reasonable that the imitation of a participant’s positive expression by a virtual agent is viewed by the participant as an automatic response and thus evokes a feeling of anthropomorphism for the virtual agent. This possibility supports our hypothesis that the imitation of positive expressions by a virtual agent is an effective way to achieve affective human–virtual agent communication.

Besides the possibility that imitation of a positive expression can activate the feeling of anthropomorphism, it is also possible that the participants had a feeling of contingency with the virtual agent, regardless of their agency belief. Since contingency can be separated from animacy^36^, the virtual agent gave positive feedback in both agency belief conditions. Taken together with the previous finding that the precuneus may be involved in updating the state of self-esteem^37^, the participants’ self-esteem could have been enhanced by the positive feedback of the virtual agent. Thus, the activation of anthropomorphism and the enhancement of self-esteem are suggested to contribute to inducing positive emotions, even in the computer belief condition. As previous studies have found, it should be noted that the medial prefrontal cortex and the precuneus are related to various factors such as episodic memory, in thinking about oneself, in thinking about the future, processing visuospatial information, and spatial attention. Thus, the psychophysiological mechanisms of inducing positive emotions by the imitation of positive expression are not limited to the feeling of anthropomorphism and the feeling of contingency^18,38–40^. Therefore, further experiments are needed to identify the dominant factor inducing a positive emotion. Our finding that none of the brain regions (including the ones found to respond more to a positive congruent response) positively tracked the level in subjective feelings should be helpful in identifying the dominant factor. This factor might even trigger the inducement of a participant’s positive emotions, rather than the factors correlated with the positive emotions.

The activated clusters also included occipital lobe areas such as the superior and middle occipital gyrus, calcarine gyrus, and lingual gyrus. One of our conjectures is that these activations were induced by an efference copy associated with a feedback connection promoting visuomotor integration^41,42^ in the smile-imitation condition. Since the timing and strength of the agent’s expressions were modulated by the degree of the participant’s smile during the smile condition, the participants received feedback on their smile through the agent’s expression. Therefore, the interaction between the effect of being imitated in the PS condition and the efference copy by the visual-motor loop could affect brain activity around the visual cortex. This possibility could be evaluated by comparison with the condition of no extent regulation of the agent’s expression based on the degree of the participant’s smile, and being imitated could be performed in accordance with whether the participant smiled or did not smile.

There are three main limitations to this study. First, only a chick-type agent with specific positive and negative expressions was used in the experiment. To our knowledge, this is the first study in which the effects of a positive expressions being imitated by a non-human virtual agent were evaluated. Further experiments and analyses are needed to clarify the key factors related to the effects of being imitated by a non-human virtual agent and to identify the basic differences between human-type and non-human-type virtual agents.

Second, a unique cover story and task instructions were used in the experiment. Whereas the participants were told that their facial expressions were going to be evaluated by an experimenter or by using AI, the actual response of the virtual agent was randomly selected. To avoid the feeling of strangeness induced by this gap, the participants were told that their affect would be estimated on the basis of their facial expressions and brain activity. Therefore, the participants were asked to try to feel happy when they smiled and to try to feel nothing when they simply looked at the virtual agent. Although this task instruction and the difficulty of feeling happy and feeling nothing contributed to filling the gap and convincing the cover story, this would have affected the subjective feelings and brain activations. In particular, case should be taken that the participants are not in a resting state when they simply looking at the virtual agent. Therefore, further experiments are needed that avoid this effect and clarify the pure effect of purely being imitated. For example, eliminating this cover story and not giving these task instuctions and simply having the virtual agent make frequent reasonable reactions (e.g. frequently making a positive response when the participants smiled) should be useful.

Finally, this study focused on the imitation of a positive expression. Previous findings that the effects of agency belief on subjective feelings, behavioral performance and brain activity can be observed in the mimicry of non-emotional responses (i.e., joint attention)^13,21^, means that the contents of imitation should be an important factor in suppressing the effect of agency belief. However, in this study, the positive emotion and brain activity associated with being imitated could have been superimposed by the effect of the positive expression itself, which helps induce a positive emotion in human-to-human communication^31,32^. Therefore, the general effect of emotion in imitation was not fully clarified. Since every (including negative) facial expression of emotion can induce the liking of the interaction partner^33^, emotion likely plays an important role in inducing positive emotions. An effective way to clarify the basic emotional effects of being imitated and to determine whether only a positive congruent response can induce positive emotions or other emotional congruent responses and/or whether neutral congruent responses can also induce positive emotions would be to have a virtual agent imitate a negative expression such as sad^43^ and/or a neutral expression such as joint attention. Thus, more work is needed to clarify the key factors in the general emotional effects of being imitated in relation to anthropomorphorism and contingency, and thereby develop affective communication between people and virtual agents.

## Methods

### Participants

Thirty-nine participants (20 men, 19 women) took part in the experiment. Since we hypothesized that the imitation of a positive expression by a virtual agent will induce a positive emotion and a feeling of anthropomorphism, regardless of the agency belief, the statistical power regarding agency belief in this study should be no lower than those in previous studies^12^. Therefore, we determined to maintain sample size at not less than those in previous studies in advance, even if data for some participants were dropped and/or excluded from the study. The average age ± SD of the participants was 21.49 ± 1.19 years old (women, 21.32 ± 0.86 years old; men, 21.65 ± 1.39 years old). All participants had normal or corrected-to-normal visual acuity. All participants were right-handed. They received monetary compensation for their time. The protocol was approved by the ethical committee of the National Institute for Physiological Sciences. Data were obtained in accordance with the standards of the internal review board on Research & Development Group, Hitachi, Ltd. The experiments were undertaken in compliance with national legislation and the Code of Ethical Principles for Medical Research Involving Human Subjects of the World Medical Association (Declaration of Helsinki). All participants provided written informed consent.

### Experimental setup

The visual stimuli were presented using a personal computer (GALLERIA GKF1060GF, ThirdWave Corp., Tokyo, Japan). A liquid crystal display projector (CP-SX12000; Hitachi, Ltd., Tokyo, Japan) located outside and behind the MRI scanner projected the stimuli through a waveguide onto a translucent screen, which the participants viewed via a mirror placed in the scanner. The spatial resolution of the projector was 1400×1050 pixels. The distance between the screen and the participant’s face was ~190 cm, and the visual angle was 13.06° (horizontal) × 10.45° (vertical). Video images of the participants’ faces were captured using an on-line grayscale video camera system (NAC Image Technology and Panasonic System Solutions Japan, Tokyo, Japan). The captured face was presented on a display, and the degree of the participant’s smile on the display was evaluated using a camera (HVC-P2, Omron, Kyoto, Japan) with facial expression evaluation software (OKAO^®^ Vision, Omron, Kyoto, Japan). Because the maximum value of the degree evaluated by the software was different for each participant, the thresholds of the agent’s expressions were individually set to match the smile on the face of each participant. The timing and strength of the agent’s expression were determined in two ways. In one case, when the participant’s smile was responded to by the agent’s positive or negative expression, the strength of the agent’s expression was determined on the basis of the threshold and the degree of the participant’s smile. Thus, the agent’s expression was triggered by the participant’s smile, and its strength was determined by the degree of the participant’s smile, making it possible to achieve a natural contingent reaction. Although the actual timing of natural or confederate mimicry is unknown^17^, it is known that a delay of more than 1 s can disrupt the feeling of being imitated^44^. Therefore, we did not intentionally set a temporal delay between the participant’s action and the agent’s response, so the agent began to respond immediately after the participants’ smile were detected. More precisely, the imitation of a positive expression was accomplished by using a combination of two cameras (a camera in the fMRI scanner room and a camera for smile evaluation); their sampling rates were 30 Hz and about 10 Hz, respectively. Therefore, the maximum temporal delay was at least 133 ms. In the other case, the timing and the strength were determined randomly. The participants’ responses (i.e., subjective feelings) were collected using an optical button box (HHSC1 × 4-D, Current Designs Inc., Philadelphia, PA, USA).

### Experimental procedure

Before entering the fMRI scanner, the participants received an explanation of the facial interaction task. Then they practiced the task at least four times inside the scanner. After becoming familiar with the task, the participants underwent fMRI scanning while performing the task. After completing the fMRI measurement, the participants completed a questionnaire for the purpose of evaluating the agency belief effect (details given in the Supplementary Fig. S1).

### Experimental task

The participants performed a facial interaction task with a non-human virtual agent in the form of a chick. In the task, the virtual agent was presented throughout a session. The participants were asked to smile (participant smile; PS) or simply look (participant look; PL) at the virtual agent when they saw a blue circle or a pink square around the virtual agent. The relationship between the participant’s facial expression (PS or PL) and figure (a blue circle or a pink square) was randomized among participants. Nineteen participants were asked to smile when they saw the blue circle, and the other 20 participants were asked to smile when they saw the pink square. The participant’s facial expression was followed by the agent producing a positive expression (AP), negative expression (ANg), or neutral expression (ANt). In addition, the AP and ANg conditions each had two strength levels (happy and very happy; sad and very sad). The agent expressions were randomly chosen and counterbalanced in each session. After this facial interaction, the participants were asked to record their feeling on a 9-point scale (from −4 to 4). A 9-point scale bar with a cursor was shown on the display, and the participants were asked to express their current feeling by moving the cursor using an optical button box within 5 s. They were also asked to move the cursor at least once when expressing their feeling. Since the initial position of the cursor was set to zero (at center), if the participants wanted to record zero, they had to move the cursor to the left or right at least once and then return it to the original position. Considering the workload and time length of the task, we did not set multiple questions for the recording feelings. The participants were simply asked to express their feeling after each trial. This procedure was explained when the participants received an explanation of the task, and they were given a chance to practice it.

The task comprised four sessions, and each session took about eight minutes. Each session contained 42 trials, 6 trials each for 6 trial types (PS_AP, PS_ANg, PS_ANt, PL_AP, PL_ANg, PL_ANt), and 6 blank trials. The order of the trial type and blank trials in each session was randomized. The durations of the pre- and post-trial rest periods in each session were both set to 14 s, and the duration of each trial and blank trial was set to 10.5 s. Each trial consisted of four periods: pre-period (0.5 s), stimulus period (4.5 s), post period (0.5 s), and rating period (5.0 s). In the stimulus period, a blue circle or a pink square around the virtual agent was presented as a signal for the participant to smile or simply look at the virtual agent. Except for the stimulus period, the virtual agent displayed a neutral expression during the trials. In other words, there was no reaction by the virtual agent in the simply look condition. Therefore, we assumed that the participants did not realize that their neutral faces were simply being mimicked by the virtual agent.

To evaluate the effect of agency belief in the task, two agency belief conditions were set; computer belief (CB) and human belief (HB). Half of the sessions were set to the CB condition, and the other half were set to the HB condition. In the CB condition, the participants were told that their affect would be estimated using artificial intelligence and that the estimated affect would be provided as the expression of the virtual agent. They were also told that they would be evaluated by an experimenter and that the evaluated affect would be provided as the expression of the virtual agent in the HB condition. The CB and HB conditions were alternately repeated twice in the experiment, and the condition order was randomly set. The first session for 19 participants was performed in the HB condition and that for 20 participants was performed in the CB condition.

In both conditions, there was a gap between the task instruction and the task design. That is, whereas the participants were told that their facial expressions were to be evaluated by an experimenter or by using AI, the actual response of the virtual agent was randomly selected. For example, while participants always smiled in the PS condition, the virtual agent positively responded once in three times on average. To avoid the feeling of strangeness induced by this gap, the participants were told that their affect would be estimated on the basis of their facial expressions and brain activity. Therefore, the participants were asked to try to feel happy when they smiled and to try to feel nothing when they simply looked at the virtual agent. This task instruction and the difficulty of feeling as instructed helped to fill the gap and convince the participants of the cover story.

To convince the participants that their facial expressions were evaluated by an experimenter in the HB condition, we fabricated a cover story and demonstrated the manually control of the virtual agent’s expressions. When the participants received an explanation of the experiment, they were told that the purpose of this study was to evaluate the use of artificial intelligence for evaluating human emotion. They were also told that the experimenter was evaluating the participants’ emotion in order to evaluate the accuracy of the AI evaluation and thereby be able to improve the accuracy of the AI evaluation. To demonstrate manual control of the virtual agent’s expressions, we developed software for manipulating the expressions in real time. Using this software, we demonstrated that the virtual agent’s expressions could be manipulated freely by keyboard input. With this combination of cover story and demonstration of manually controlling of the virtual agents’ expressions, we convinced the participants that the virtual agent was controlled by the experimenter, thereby creating the HB condition.

### MRI data acquisition

A 3 T MRI scanner (Magnetom Verio 3 T, Siemens Medical Systems, Erlangen, Germany) was used to obtain whole-brain functional images. This study used a 32-channel phased array coil modified to consist of 24 channels, as was done in a previous study by our group^45^. The Siemens Verio standard 32-channel phased array coil consists of a bottom component with 20 channels and a top component with 12 channels. Since the top component of the coil covers part of the face, it was unsuitable for evaluating the participant’s smile. Therefore, it was replaced with a small four-channel flex coil (Siemens) that was attached with a special holding fixture (Takeshima Seisakusho Co., Tokyo, Japan). We used sparse sampling with a T2*-weighted echo planar imaging (EPI) gradient-echo sequence (echo time [TE] = 35 ms; repetition time [TR] = 3500 ms; acquisition time [TA] = 500 ms; field of view [FoV] = 192 × 192 mm; flip angle = 58°; matrix size = 64 × 64; 42 slices; slice thickness = 3 mm; total number of volumes = 537). Six slices were acquired simultaneously using a multiband sequence^46^. A whole brain, high-resolution, T1-weighted anatomical MR image acquired using a magnetization-prepared rapid acquisition gradient-echo (MP-RAGE) sequence was used for anatomical localization with the standard 32-channel phased array coil (TE = 2.24 ms; TR = 2400 ms; FoV = 256 × 256 mm; flip angle = 8°; matrix size = 300 × 324; slice thickness = 0.8 mm).

### Behavioral data analysis

We used SPSS ver.20 to analyze the behavioral data. The behavioral data in a trial without any cursor movement were filtered out. Because there was no significant correlation between expression strength and subjective feeling, the average values of the subjective feelings across the strengths in each condition were used. A Kolmogorov-Smirnov normality test using ensemble averaged behavioral data produced no significant results, so the ensemble-averaged data were regarded as normally distributed. To examine the effects of participant action, agent reaction, and agency belief and their interactions on participant subjective feeling, we performed a three-way ANOVA with post hoc Bonferroni tests. When the main effect of one factor or the interaction between factors was significant, paired *t*-tests were performed as post hoc tests. The significance level for multiple comparison in these tests was corrected by using the Bonferroni method. In cases where Mauchly’s sphericity test was significant, the Greenhouse-Geisser correction was applied. The significance level was set at *p* < 0.05.

### fMRI data analysis

We used SPM12 version 6685 (The Wellcome Trust Centre for NeuroImaging; https://www.fil.ion.ucl.ac.uk/spm/) implemented in MATLAB R2017b (MathWorks, Inc., Massachusetts, USA) to analyze the functional images. The first four volumes of each fMRI session were discarded because the MRI signal was unsteady. We performed head motion correction on the remaining volumes by realignment, coregistration of functional and structural images, and normalization to the Montreal Neurological Institute (MNI) template. Then, anatomically normalized EPI images were then resampled to a voxel size of 2 mm × 2 mm × 2 mm and spatially smoothed using a Gaussian kernel of 8 mm full-width at half maximum.

In a first level analysis, the evaluation conditions were classified on the basis of the combination of the participant’s expression (smile: PS, simply look at the agent: PL) and the agent’s expression (positive expression: AP, negative expression: ANg, neutral expression: ANt). We defined six regressors of interest [the condition that PS was followed by AP (PS_AP), ANg (PS_ANg), and ANt (PS_ANt), PL was followed by AP (PL_AP), ANg (PL_ANg), and ANt (PL_ANt)] and nine regressors of no interest (ROIs) [ROIs of white matter and cerebrospinal fluid (CSF) volume, subjective rating, and representative motion parameter]. The ROIs of white matter and CSF volume were extracted using MarsBaR software^47^. Then all explanatory variables, except for three nuisance parameters (ROIs of white matter and CSF volume and a movement parameter), were convolved with a canonical hemodynamic response function and entered into a general linear model. The duration of each trial for the regressors was 4.5 s. To reveal the neural substrate of the positive expression imitation by the virtual agent, the contrast was set to (PS_AP − (PS_ANg + PS_ANt)/2) × (PL_AP − (PL_ANg + PL_ANt)/2). The contrast was then entered into group analysis using a one-sample *t*-test. To determine the effect of agency belief (computer belief: CB; human belief: HB), we calculated both CB > HB and CB < HB in the contrast of (PS_AP − (PS_ANg + PS_ANt)/2) − (PL_AP − (PL_ANg + PL_ANt)/2).

The statistical threshold for activation of cluster formation was set at *p* < 0.001 uncorrected, and for cluster-level family-wise error (FWE) corrected it was set at *p* < 0.05. Anatomical labeling was based on Automated Anatomical Labeling^48^ and the Anatomy toolbox v2.1^49^.

With the above information, we report how we determined our sample size, all data exclusions (if any), all manipulations, and all measures in the study.

## Supplementary information

Supplementary information.

## Data Availability

The datasets generated during the current study are available from the corresponding author on reasonable request.
