# Supplementary material for: Achieving affective human–virtual agent communication by enabling virtual agents to imitate positive expressions
Source: Sci Rep. 2020 Apr 6;10:5977. doi: 10.1038/s41598-020-62870-7 (PMC7136238; doi:10.1038/s41598-020-62870-7)
Supplement: Supplementary file 1 — Supplementary information. [file 41598_2020_62870_MOESM1_ESM.docx]

**Title**

Achieving affective human–virtual agent communication by enabling virtual agents to imitate positive expressions

**Author affiliations**

Takashi Numata^1,*^, Hiroki Sato^2^, Yasuhiro Asa^1^, Takahiko Koike^3^, Kohei Miyata^3^, Eri Nakagawa^3^, Motofumi Sumiya^3^, Norihiro Sadato^3^

^1^Center for Exploratory Research, Research & Development Group, Hitachi, Ltd., Hatoyama, Saitama, 350-0395, Japan

^2^Department of Bioscience and Engineering, Shibaura Institute of Technology, Saitama, Saitama, 337-8570, Japan

^3^Division of Cerebral Integration, Department of System Neuroscience, National Institute for Physiological Sciences, Okazaki, Aichi, 444-8585, Japan

Correspondence and requests for materials should be addressed to T. N.

(email: takashi.numata.rf@hitachi.com)

**Supplementary information**

We supplementary show the results of subjective feeling ratings by participant expression (Smile, Simply Look), agent response (Positive, Negative, Neutral), and agency belief (Computer, Human), during the interaction task (Table S1). In addition, we show the results of comparison of subjective ratings between human and computer belief conditions, after the interaction task (Figure S1).

Animations of the virtual agent in this study are available from the corresponding author on reasonable request.

Table S1 **Behavioral results**.

| Participant’s  Expression | Smile (PS) | | | Simply look (PL) | | |
| --- | --- | --- | --- | --- | --- | --- |
| Agent’s reaction | Positive (AP) | Negative (ANg) | Neutral (ANt) | Positive (AP) | Negative (ANg) | Neutral (ANt) |
| Human belief | 2.01 ± 0.89 | -0.86 ± 1.16 | -0.48 ± 1.23 | 0.18 ± 1.08 | -0.56 ± 0.88 | -0.05 ± 0.45 |
| Computer belief | 2.06 ± 0.93 | -0.88 ± 1.30 | -0.46 ± 1.33 | -0.00 ± 1.09 | -0.66 ± 0.79 | -0.10 ± 0.47 |

Subjective feeling ratings by participant expression (Smile, Simply Look), agent response (Positive, Negative, Neutral), and agency belief (Computer, Human). Statistical results are shown in Fig. 3. Numbers that follows the ± sign are standard deviations (s.d.).

**Figure S1. Comparison of subjective ratings between human and computer belief conditions.** These subjective ratings were obtained after all sessions had been completed, outside the fMRI scanner, using a visual analogue scale (VAS) on which “strongly agree” was the right-most rating and “strongly disagree” was the left-most rating (with five ratings in between). The subjective ratings were calculated in the range from −50 to 50. The rating descriptions on the scale and the questionnaire items were written in Japanese. A paired t-test with Bonferroni correction revealed no significant differences among the seven subjective ratings. The items on the questionnaire were (A) “The chick-type CG agent demonstrated human-like reactions to my facial expressions,” (B) “the chick-type CG agent demonstrated natural reactions to my facial expressions,” (C) “the chick-type CG agent was likeable,” (D) “the chick-type CG agent was reliable,” (E) “the chick-type CG agent was friendly,” (F) “the chick-type CG agent empathized with me,” and (G) “I would like to use a communication application with a chick-type CG agent.”
